# The Effect of Goat-Milk-Based Infant Formulas on Growth and Safety Parameters: A Systematic Review and Meta-Analysis

**DOI:** 10.3390/nu15092110

**Published:** 2023-04-27

**Authors:** Mateusz Jankiewicz, Linde van Lee, Mirthe Biesheuvel, Elske M. Brouwer-Brolsma, Lucie van der Zee, Hania Szajewska

**Affiliations:** 1 Department of Paediatrics, The Medical University of Warsaw, 02-091 Warsaw, Poland; mateusz.j.jankiewicz@gmail.com; 2 Ausnutria B.V., 8021 Zwolle, The Netherlands; 3 Division of Human Nutrition & Health, Wageningen University, 6705 Wageningen, The Netherlands

**Keywords:** infant, children, infant formula, goat, nutrition, feeding, growth

## Abstract

Goat-milk-based infant formulas (GMFs) are now available in several countries, having been approved by authorities. We systematically evaluated the effects of GMF compared with cow-milk-based formula (CMF) on infant growth and safety parameters. The MEDLINE, EMBASE, and Cochrane Library databases were searched (December 2022) for randomized controlled trials (RCTs). The risk of bias was assessed using the Revised Cochrane Risk-of-Bias tool (ROB-2). Heterogeneity was quantified by *I*^2^. Four RCTs involving a total of 670 infants were identified. All trials revealed some concern in ROB-2. Furthermore, all of the included studies were funded by the industry. Compared with infants fed CMF, those fed GMF showed similar growth in sex- and age-adjusted *z*-scores for weight (mean difference, MD, 0.21 [95% confidence interval, CI, −0.16 to 0.58], *I^2^* = 56%), length (MD 0.02, [95% CI −0.29 to 0.33], *I^2^* = 24%), and head circumference (MD 0.12, 95% [CI −0.19 to 0.43], *I^2^* = 2%). Stool frequency was similar among the groups. Due to differences in the reporting of stool consistency, no firm conclusion can be drawn. Adverse effects (serious or any) were similar in both groups. These findings provide reassurance that GMFs compared with CMFs are safe and well tolerated.

## 1. Introduction

Early life nutrition substantially impacts growth and long-term health, particularly during the first months of life when major developments unfold within the central nervous system, gastrointestinal tract, and immune system [1]. According to the World Health Organization (WHO), exclusive breastfeeding for the first 6 months of life is the preferred feeding option [2]. Human milk is rich in nutrients, bioactive compounds, and immune factors, which promote optimal growth and development and protect against infections and chronic diseases. Breastfeeding also has benefits for the mother, including reduced risk of breast and ovarian cancer [3]. However, the proportion of infants worldwide who are breastfed in accordance with the guidelines set by the WHO remains suboptimal, with less than 50% meeting these guidelines [4,5,6]. Therefore, effective strategies to protect, promote, and support breastfeeding are still needed. In the meantime, infants who cannot be breastfed, or should not receive breast milk, or for whom breast milk is not available, require breast milk substitutes of high quality [7].

Cow-milk-based formulas (CMFs) are commonly used as breast milk substitutes, and their quality has significantly improved over the years. At the same time, the increasing global production of goat milk, which requires lower feeding requirements, shorter generation intervals, and lower production costs compared to cow’s milk [8,9], has prompted research into the use of goat milk as a basis for infant formula. Goat milk is expected to have several health benefits, such as easier digestion [10].

Compared with cow milk, goat milk has a higher level of α_S2_-casein, which is linked to forming smaller flocs of aggregated protein that were digested more effectively in an in vitro infant gastric model [11,12]. In a comparative study on rodents, goat milk increased the rate of gastric emptying as compared to cow milk, probably due to the coagulation properties of these two milks [13]. As the capacity of an infant’s stomach is less than that of an adult, the gastric digestion behaviors of infant formulas are of essence [14].

In some countries, goat-milk-based infant formulas (GMFs) are available and have been approved by agencies such as the European Food Safety Authority (EFSA) [15]. Addtionally, at least one manufacturer has been granted a generally recognized as safe (GRAS) status for their nonfat dry goat milk and goat why protein concentrate for use in infant formulas [16]. Since the regulatory assessments, new evidence has become available. While it is crucial to prioritize effective strategies for promoting, protecting, and supporting breastfeeding, it is also important to provide evidence-based guidance to healthcare professionals and policymakers. This guidance can help optimize infant feeding practices in cases where breast milk is not available. Therefore, this systematic review aims to evaluate the effects of feeding infants GMF compared with CMF on growth and safety parameters.

## 2. Materials and Methods

This systematic review and meta-analysis was conducted following the guidelines from the Cochrane Handbook for Systematic Reviews of Interventions [17] and reported according to the Preferred Reporting Items for Systematic Reviews and Meta-Analyses (PRISMA) statement [18]. However, the protocol was not registered. Ethical approval was not needed.

### 2.1. Eligibility Criteria

Studies eligible for inclusion had to be randomized controlled trials (RCTs) conducted in healthy term infants with reported exposure to GMF in the intervention group and CMF in the control group. At inclusion, GMF had to be the only source of nutrition in the intervention group, and CMF in the controls. The included trials had to be published as full texts, with no language or publication date restriction. The eligible outcomes had to include at least one measure of growth (weight, length, head circumference, body mass index, WHO indices/*z*-scores), at least one measure of safety (adverse events, tolerability, biomarkers), or at least one measure of stool characteristics (e.g., stool frequency, stool consistency). Other reported outcomes were considered if relevant to the current review. Studies conducted in infants who were preterm (gestation < 37 weeks), with a low birthweight (<2.5 kg), and/or diagnosed with a severe illness likely to affect growth (e.g., congenital or metabolic diseases, infections, or allergy) were excluded.

### 2.2. Search Strategy

The MEDLINE (PubMed), EMBASE, and the Cochrane Central Register of Controlled Trials (CENTRAL, the Cochrane Library) databases were searched using the pre-specified search strategy. Three search strategies were developed (Supplementary Materials, Table S1). The search was performed twice (October 2020 and a repeated in December 2022). Additionally, references from other relevant review articles were screened for RCTs not identified by the primary search. The ClinicalTrials.gov and ClinicalTrialsRegister.eu websites were also searched for RCTs that were registered but not yet published.

### 2.3. Selection of Studies

Two reviewers independently screened the titles and abstracts of the identified studies. Subsequently, full texts of potentially relevant articles, as well as studies with unclear relevance, were acquired and individually reviewed for eligibility by two other reviewers. Eligibility was assessed with a standardized full text screening form. Any disagreement among the reviewers was resolved through discussion.

### 2.4. Data Extraction and Management

Data were extracted using a predefined data extraction form, which included the trial year, country, population, intervention, comparison, outcomes, results, data collection methods, sample size calculation, availability of study protocol, and funding. For each trial, the second reviewer checked the completeness and accuracy of the extracted data.

### 2.5. Assessment of Risk of Bias in Included Studies

For each trial included, the risk of bias was assessed using the second version of the Cochrane Collaboration tool (ROB-2) [19] by two independent assessors. Five domains were assessed: bias arising from the randomization process, bias due to deviations from the intended interventions, bias from missing outcome data, bias in measurement of the outcome, and bias in selection of the reported result with one of the signaling questions (yes, probably yes, probably no, no, and no information). For each individual and domain and for the overall judgment, the trials were classified into low risk of bias, some concerns, or high risk of bias. Any disagreements were resolved through discussion with other reviewers who were not the assessors.

### 2.6. Data Collection and Analysis

For dichotomous outcomes, the relative risk (RR) between the experimental and control groups with 95% confidence intervals (CI) was reported. Continuous data were reported as mean differences (MD) with 95% CI, with extracted mean values, standard deviations (SDs), and number of participants in outcomes. Heterogeneity was assessed with the use of χ2 and *I*^2^ tests. For the latter, a value of 0% to 40% might not be important; 30% to 60% may represent moderate heterogeneity; 50% to 90% may represent substantial heterogeneity; and 75% to 100% indicates considerable heterogeneity [17]. All analyses were based on the random-effects model. The data were analyzed with the use of Review Manager (RevMan) (Computer program. Version 5.4. The Cochrane Collaboration, 2020). If data were reported as the mean with CI, SDs were calculated using the built-in calculator in RevMan. The publication bias was not assessed, as sufficient (≥10) eligible trials were not available. For completeness, we also present the results of comparisons of the GMF group with a non-randomized reference group fed human breast milk.

## 3. Results

### 3.1. Characteristics of Included Studies

**Figure 1:**
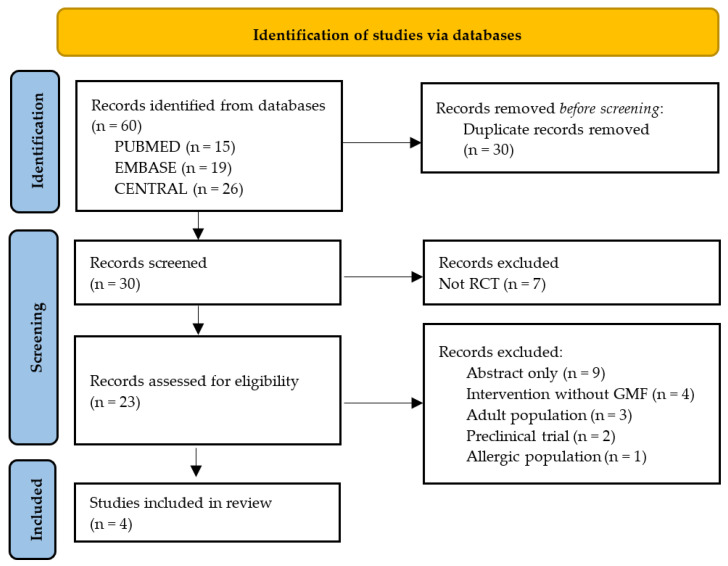
Flow diagram. RCT, randomized controlled trial; GMF, goat milk formula.

The search strategy generated 60 records in the databases. The flow diagram of the selection process is presented in Figure 1.

Four RCTs met the inclusion criteria [20,21,22,23]. A total of 670 infants participated in the included trials with a median of 148.5 participants per trial (range: 72–301). The median intervention period was 175.5 days (range: 112–365). Two trials [21,23] recruited newborns at 14 days of age; one trial recruited infants younger than 72 hours of age; and one trial recruited infants younger than 3 months of age. Each study’s primary outcomes included weight, length, and head circumference at different time points. All of the included studies used GMF in the intervention group and were controlled with CMF. In two trials [21,23], there was also a non-randomized breastfed group that received human milk (HM). For details of the included trials, see Table 1. In addition, two RCTs registered in ClinicalTrials.gov (accessed on 27 February 2023) were identified, including one trial evaluating the effects of a goat milk infant formula on the risk of allergy in the first 5 years of life (NCT04599946; Goat Infant Formula Feeding and Eczema (the GIraFFE Study)) and one trial evaluating gastrointestinal regurgitation (NCT05363553; TIGER Study). However, these trials are recruiting participants, and the results have not been published yet.

### 3.2. Risk of Bias Assessment

**Figure 2:**
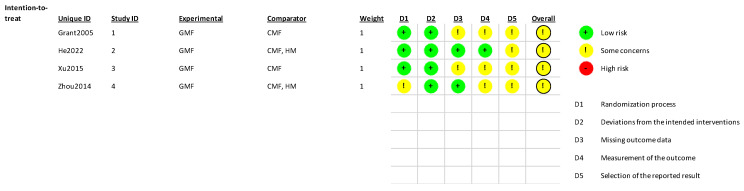
Risk of bias assessment. GMF, goat-milk-based formula; CMF, cow-milk-based formula; HM, human milk, Grant 2005 [20], He 2022 [23], Xu 2015 [22], Zhou 2014 [21].

Overall, the risk of bias was assessed as raising some concern in the four included RCTs. The most common concerns were related to the selection of reported results (four trials), measurement of the outcome (three trials), missing outcome data (two trials), and randomization process (one trial). For details, see Figure 2.

### 3.3. Findings

**Figure 3:**
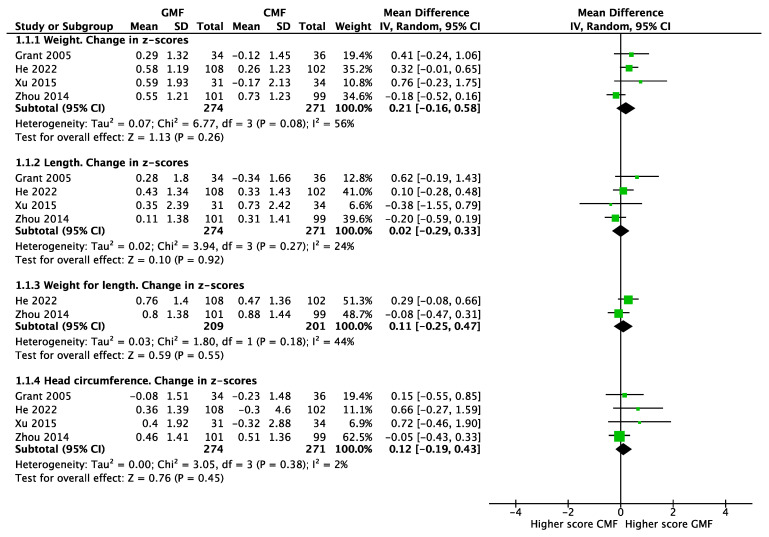
Anthropometrics of infants fed goat-milk-based formula (GMF) compared to cow-milk-based formula (CMF), Grant 2005 [20], He 2022 [23], Xu 2015 [22], Zhou 2014 [21].

*Anthropometrics*. Based on the pooled results of four RCTs involving 545 infants [20,21,22,23], there were no significant differences between infants fed with GMF or CMF in the mean changes in *z*-scores for weight (MD 0.21; 95% CI −0.16 to 0.58; *I*^2^ = 56%), length (MD 0.02; 95% CI −0.29 to 0.33; *I*^2^ = 24%), and head circumference (MD 0.12; 95% CI −0.19 to 0.43; *I*^2^ = 2%). The pooled results of two RCTs [21,23], involving 410 infants, showed no statistically significant differences between groups in the mean changes in weight-for-length *z*-scores (MD 0.11; 95% CI −0.25 to 0.47; *I*^2^ = 44%) (Figure 3).

**Figure 4:**
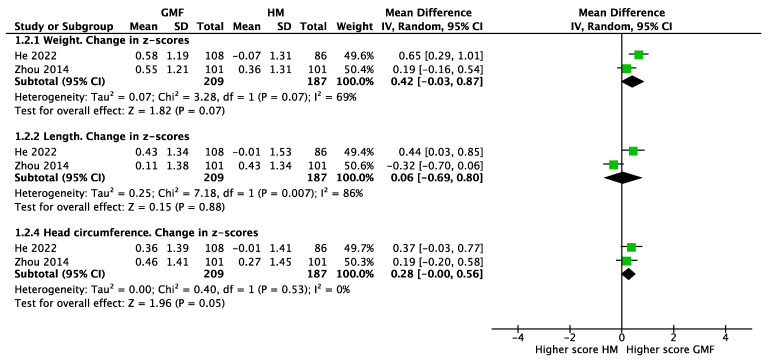
Anthropometrics of infants fed goat-milk-based formula (GMF) compared to human milk (HM), He 2022 [23], Zhou 2014 [21].

The comparison of infants fed GMF with a non-randomized reference group fed human milk found no differences in the mean changes in *z*-scores for weight (two studies, *n* = 396, MD 0.42; 95% CI −0.03 to 0.87; *I*^2^ = 69%), length (MD 0.06; 95% CI −0.69 to 0.80; *I*^2^ = 86%), and head circumference (MD 0.28; 95% CI −0.00 to 0.56; *I^2^* = 0%) (Figure 4).

**Figure 5:**
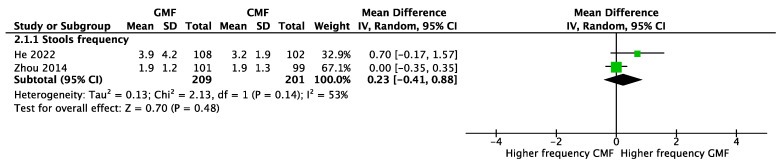
Stool frequency of infants fed goat-milk-based formula (GMF) compared to cow-milk-based formula (CMF), He 2022 [23], Zhou 2014 [21].

**Figure 6:**
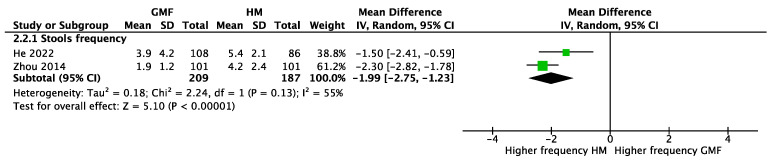
Stool frequency of infants fed goat-milk-based formula (GMF) compared to human milk (HM), He 2022 [23], Zhou 2014 [21].

*Stools.* Two RCTs [21,23] involving 410 participants reported no significant difference between GMF-fed and CMF-fed infants in stool frequency (MD 0.23; 95% CI −0.41 to 0.88; *I^2^* = 53%) (Figure 5). However, infants receiving GMF compared with human milk had a lower stool frequency (two studies, *n* = 396, MD −1.99; 95% CI −2.75 to −1.23; *I*^2^ = 55%) (Figure 6).

The four included studies [20,21,22,23] differed in the methodology of stool consistency assessment. One study [21] involving 301 participants reported significantly lower mean (SD) scores on the Bristol Stool Scale [24] in GMF-fed compared to CMF-fed infants at 2 weeks, 4.69 (1.44) versus 5.46 (0.96), and at 1 month, 4.59 (1.35) versus 5.35 (1.19), respectively. The Bristol Stool Scale refers to seven pictures of different forms of stool (from 1 to 7; types 3, 4, and 5 are considered normal stool forms). One study [23] involving 218 participants reported a significantly higher mean (SD) score on the Infant Stool Form Scale (the “Amsterdam” scale) [25] in the GMF-fed group compared to the CMF-fed group of 2.2 (0.6) versus 2.0 (0.4), respectively. Among others, the “Amsterdam” scale enables evaluation of stool consistency (watery, soft, formed, hard); in the study, lower scores indicated more watery stools.

One study [20] reported no significant differences in “runny” and “hard” bowel motions between groups, although the methods of assessment remain unclear (Table 2).

*Other outcomes.* One RCT [21] involving 301 participants reported no differences in food allergy between GMF-fed and CMF-fed infants (2/92 and 1/89, respectively). There was also no difference in atopic dermatitis using SCORing Atopic Dermatitis (SCORAD) between the groups (13/91 and 20/86, respectively) and in the mean (SD) SCORAD (9.9 (6,7) and 11.9 (7.1), respectively). A comparison of infants fed GMF with a non-randomized reference group fed human milk found no differences in food allergy or atopic dermatitis.

*Serious adverse events.* Based on four RCTs [20,21,22,23] involving 670 participants, no differences were found in the frequency of adverse events (serious or any) between GMF-fed and CMF-fed infants (RR 0.83; 95% CI 0.52 to 1.35; *I^2^* = 11%). Among reported serious adverse events, the most frequent were respiratory tract infections (with its manifestations: runny nose, bronchiolitis, fever) and gastrointestinal symptoms (mainly diarrhea) (Table 3).

## 4. Discussion

This systematic review of four RCTs summarizes the current evidence on the potential impact of GMF compared with CMF on infant growth, stool frequency and consistency, and adverse effects. Our findings show no significant differences in anthropometric parameters or stool frequency between infants fed with GMF compared to CMF. While no firm conclusion can be drawn regarding stool consistency due to the variety of assessment methods used across the four studies, the stool scores suggest that infants fed GMF may have more solid stools, which are still considered normal. Adverse events were similar in both groups.

There was no difference in anthropometric parameters and in food allergy or atopic dermatitis between GMF-fed infants and both CMF-fed infants and non-randomized breastfed infants. GMF-fed infants did exhibit a tendency toward lower stool frequency. However, these findings are based on a non-randomized group comparison and are presented for completeness only.

### 4.1. Strengths and Limitations

While there are strengths to this systematic review and meta-analysis, such as the use of rigorous methodology developed by the Cochrane Collaboration, a comprehensive literature search with no language restrictions, and pre-specified criteria for methodological assessment and analysis, there are also limitations. One limitation is that we did not publish the protocol of the review, which limits the transparency and validity of the study methods. Furthermore, the limited number of RCTs identified reduces statistical power, limits generalizability, and makes it difficult to identify reporting bias. Additionally, the methodological quality of the included trials varied, and all studies were judged as presenting some concerns with regard to the risk of bias. This is an important consideration, as bias can affect the reliability and validity of study findings. While the assessment of growth parameters was made based on well-established scientific principles (including sample size calculation and duration of the follow-up), the reporting of other outcomes varied (e.g., stool consistency). Finally, all included studies were industry-supported, and many of the co-authors of these studies are employed by the manufacturers of GMF. This could potentially introduce bias, as industry-supported studies have been shown to be more likely to report positive findings [26]. Overall, all the limitations underscore the need for caution when interpreting the results of the review.

### 4.2. Agreement and Disagreement with Other Studies

Our systematic review builds upon earlier narrative reviews, which examined the properties of goat milk and cow milk as a base for infant formula [27], as well as the nutritional and potential beneficial features of goat milk for commercial products [28]. Both reviews indicated the potential benefits of goat milk as a source of proteins and fatty acids for dairy products.

Our review focused on RCTs. However, observational studies have reported some evidence that GMF administered to infants contributes to the improvement of stool characteristics and a reduction in some gastrointestinal symptoms. For instance, a case series study found that GMF was associated with an improvement in stool characteristics [29]. After 3 weeks of intervention, infants fed with GMF had higher scores (softer stools) on the Bristol Stool Scale. However, the number of stools per day was not significantly different. The same study also showed a reduction in total crying time per 24 h, from 3 h at inclusion to 1 h after 3 weeks, as reported by the parents. Parent satisfaction was also measured using a Likert-type scale; at the second visit, all parents were “satisfied” or “very satisfied” (*n* = 20). At inclusion, parents reported various answers, with 11 being “very dissatisfied” or “dissatisfied”, 3 being “neutral”, and 6 being “satisfied” or “very satisfied”. However, both parents and outcome assessors were not blinded to the intervention, and there was also no parallel control group.

Another study [30] reported an improvement in the Cow’s Milk-related Symptom Score (CoMiSS) in infants fed with GMF. The CoMiSS is a combined score assessing symptoms from five domains: crying, regurgitation, stools, skin, and respiratory. The study found a 50–75% reduction in CoMiSS after the intervention period in GMF-fed infants. Before introducing GMF, cow milk products were eliminated from the infants’ diet for 3 weeks.

A prospective cohort of 976 infants from birth to 12 months of age [31] found no significant differences in weight gain between infants fed with GMF or a combination of HM and GMF compared to infants fed with CMF or a combination of HM and CMF during the first 4 months of life. However, the study did report differences in bowel motions among the infants. Infants fed with GMF had more frequent stools than CMF-fed infants, with the average number of stools per day being similar for GMF-fed and HM-fed groups. The CMF-fed group was more likely to have one–two stools per day, while the GMF-fed group was more likely to have seven or more stools per day. The consistency of stools in the CMF-fed group was firmer than that in the HM-fed and GMF-fed groups. It is important to note, though, that this study was unblinded for outcome assessors and parents, and the allocation was chosen by the mothers.

Overall, while the findings from the observational studies are of interest, their conclusions need to be interpreted with caution due to the methodological shortcomings and limitations, such as potential bias and confounding. It is important to determine whether such effects will be confirmed in an RCT.

The potential benefits of GMF feeding in infancy remain unclear. The upcoming studies may play a role in exploring the potential advantages of goat milk as a source of fat or proteins for infant formulas. The GIraFFE Study (NCT04599946) will explore the impact of GMF on atopic dermatitis. This trial will determine the effect of GMF or CMF feeding in the first year of life on the risk of allergy and other health outcomes, including growth, tolerance, and quality of life in the first 5 years of life. GMF is also expected to be digested more effectively than CMF, as suggested by preclinical studies [11,12,13]. These features are linked with the anatomy and physiology of the infant’s stomach. Another upcoming trial (NCT05363553; TIGER Study) will explore the effect of GMF or CMF feeding in the first 6 months of life on the prevalence of functional gastrointestinal disorders. Among the outcomes are the frequency of infant regurgitation, infant colic, and functional constipation (diagnosed with Rome IV criteria), as well as stool frequency and feeding difficulties.

## 5. Conclusions

The findings from published trials provide reassurance that the GMFs compared with CMFs evaluated are safe and well tolerated by infants who cannot be breastfed. However, it is important to note that the current evidence base is not yet sufficient to definitively conclude whether GMFs offer clear benefits for health outcomes compared to CMFs. Therefore, healthcare providers should carefully consider the individual needs and circumstances of each infant and family when recommending a feeding option. Further RCTs are needed to better understand the potential advantages and disadvantages of GMFs compared with CMFs and to inform clinical practice. It is worth noting that breastfeeding remains the natural and optimal nutrition for infants, and healthcare providers should encourage and support breastfeeding whenever possible.

## Figures and Tables

**Table 1:** Included studies.

Author, Year [Reference]	Age at Enrollment	Participants Included (Analyzed)	Formula Feeding at Enrollment	Data Collection Methods	Intervention * (Intake Mean, SD) $ (Intake Median, Q1–Q3)	Control * (Intake Mean, SD) $ (Intake Median, Q1–Q3)	Follow-Up	Outcomes	Published Study Protocol Available (Yes/No)	Sample Size Calculation (Yes/No)	Funding
Grant et al., 2005, New Zealand [20]	Birth to 72 h	72 (70)	Exclusive formula feeding	Parent reporting diary, anthropometric measurements	* GMF (820, 133) mL	* CMF (865, 125) mL	Until 168 days of age	Weight, length, HC at 14, 28, 56, 84, 112, 140, and 168 days; bowel motion frequency and consistency; sleeping and crying patterns, adverse events	No	Yes	Dairy Goat Co-operative (N.Z.) Ltd, Hamilton
Zhou et al., 2014, Australia [21]	Birth to 14 days	200 (200) plus 101 who were BMF	Exclusive formula feeding	Blood sample, parent reporting form, anthropometric measurements	GMF $ (698, 570–825) mL at first 2 weeks ^1^ $ (1000, 855–1190) mL at 4 and 6 months ^1^	CMF BMF $ (698, 570–825) mL at first 2 weeks ^1^ $ (1000, 855–1190) mL at 4 and 6 months ^1^	Until 4–12 months of age	Weight, length, HC at 2 wk and 1, 2, 3, 4, 6, and 12 months of age, reported as z-scores; nutritional status (serum albumin, urea, creatinine, hemoglobin, ferritin, folate, plasma amino acid concentration at 4 months); health problems (respiratory illness, gastrointestinal illness, reflux, eye infection, ear, nose, and throat conditions, fever, urinary tract infection, and thrush); SAE (death or hospital admission for more than 24 h); dermatitis (SCORAD); symptoms related to food allergy and/or GI function; stool frequency/consistency; sleep patterns	Yes	Yes	Dairy Goat Co-operative (N.Z.) Limited, New Zealand
Xu et al., 2015, China [22]	0–3 months	79 (65)	Not exclusive formula feeding	anthropometric measurements, parent reporting diary, blood samples	GMF * (76.88, 43.98) g at enrollment ^1^ * (173.40, 71.35) g after 6 months of intervention ^1^	CMF * (76.88, 43.98) g at enrollment ^1^ * (173.40, 71.35) g after 6 months of intervention ^1^	6 months	Weight, length, and HC, measured at enrollment, 3 months, and 6 months, reported as z- scores; health problems, including respiratory illness, gastrointestinal illness, reflux, eye infection, ear, nose, and throat conditions, fever, urinary tract infection, and thrush; SAE (death or hospital admission for more than 24 h); blood elements (Ca, Mg, Fe, Zn, Cu levels in serum); urinal and fecal parameters	No	Yes	GMF and CMF were manufactured and provided by Ausnutria Hyproca Dairy Group BV Financially supported by Beijing Municipal Science & Technology Commission
He et al., 2022, The Netherlands [23]	0–14 days	218 (153) plus 86 (75) who were BMF	Exclusive formula feeding	Anthropometric measurements, blood and stool samples, parent reporting form	GMF * (765.8, 225.2) mL at day 14 * (953.8, 257.1) mL at day 112	CMF * (781.4, 157.2) mL at day 14 * (985.5, 242.5) mL at day 112 BMF (Not randomized)	112 d	Weight, length, and HC at baseline and at 14, 28, 56, 84, and 112 study days; stool characteristics (infant stool form scale); tolerability symptoms (reflux, colic, flatulence, and fussiness); medication use; AE (any untoward medical occurrence in a subject during the study period); SAE (death, hospitalization, or prolongation of existing hospitalization, persistent or significant disability or incapacity, or an important medical event)	Yes	Yes	Ausnutria B.V.

* (intake, mean, SD); $ (intake, median, Q1–Q3), GMF, goat milk formula; CMF, cow milk formula; SAE, serious adverse event; AE, adverse event; HC, head circumference; BMF, breastmilk-fed. ^1^ Intake median was calculated and includes GMF and CMF groups in grams of powder.

**Table 2:** Stool characteristics.

Study	Assessed Outcome	Stool Characteristics		
		Goat Milk Formula	Cow Milk Formula	Human Milk
Grant 2005 [20]	Bowel motions per day Median (5th, 95th centiles)	2.4 (1.1, 4.0) *	1.7 (1.0, 4.4) *	-
	Runny bowel motions at any visit, *n* (%)	5 (15)	6 (17)	-
	Hard bowel motions at any visit, *n* (%)	4 (12)	2 (6)	-
Zhou 2014 [21]	Stool motions per day, mean (SD)			
	2 weeks	2.5 (1.6)	2.5 (1.4)	6.3 (3.3) ***
	1 month	2.0 (1.3)	2.0 (1.4)	5.0 (2.3) ***
	2 months	1.6 (1.0)	1.5 (0.9)	3.0 (2.2) ***
	3 months	1.6 (0.9)	1.6 (1.3)	2.4 (1.8) ***
	Stool consistency in BSS, mean (SD)			
	2 weeks	4.69 (1.44) ****	5.46 (0.96) ****	-
	1 month	4.59 (1.35) **	5.35 (1.19) **	-
	Other time points were not reported			
Xu 2015 [22]	Not reported			
He 2022 [23]	Stool consistency in IFSF, mean (SD)	2.2 (0.6) ***	2.0 (0.4) ***	1.8 (0.5) ***

Original nomenclature was used, BSS = Bristol Stool Scale, ISFS = Infant Stool Form Scale. * *p* < 0.05, ** *p* < 0.01, *** *p* < 0.001, **** *p* < 0.0001.

**Table 3:** Frequency of serious adverse events.

Study	Intervention	Total Included	Any SAEs	Bronchiolitis	Fever and Cough	Cough	Viral Meningitis	Epistaxis	Pneumonia	Pallor with High Heart Rate	Febrile Illness	Strangulated Hernia	Accidentally Dropped	Diarrhea	Throat Conditions	Runny Nose	Eczema	Infections/Infestations	GI Disorders	Metabolism and Nutrition Disorders
Grant 2005 [20]	GMF	36	5	2	1	1	1	-	-	-	-	-	-	-	-	-	-	-	-	-
	CMF	36	7	-	1	-	-	1	1	1	1	1	1	-	-	-	-	-	-	-
Zhou 2014 [21]	GMF	101	15	-	-	-	-	-	-	-	-	-	-	-	-	-	-	-	-	-
	CMF	99	12	-	-	-	-	-	-	-	-	-	-	-	-	-	-	-	-	-
	HM	101	9	-	-	-	-	-	-	-	-	-	-	-	-	-	-	-	-	-
Xu 2015 [22]	GMF	39	6	-	-	-	-	-	-	-	-	-	-	2	4	-	-	-	-	-
	CMF	40	7	-	-	-	-	-	-	-	-	-	-	3	2	1	1	-	-	-
He 2022 [23]	GMF	108	5	-	-	-	-	-	-	-	-	-	-	-	-	-	-	2	1	1
	CMF	102	12	-	-	-	-	-	-	-	-	-	-	-	-	-	-	6	2	-
	HM	86	4	-	-	-	-	-	-	-	-	-	-	-	-	-	-	1	1	-

AEs = adverse events; SAEs = serious adverse events; GMF = goat milk formula; CMF = cow milk formula; HM = human milk; GI = gastrointestinal.

## Data Availability

All data are presented within the manuscript text and tables.

## Supplementary Materials

The following supporting information can be downloaded at: https://www.mdpi.com/article/10.3390/nu15092110/s1, Table S1. Search strategy.
