# Supplementary material for: The Effect of Goat-Milk-Based Infant Formulas on Growth and Safety Parameters: A Systematic Review and Meta-Analysis
Source: Nutrients. 2023 Apr 27;15(9):2110. doi: 10.3390/nu15092110 (PMC10181279; doi:10.3390/nu15092110)
Supplement: Supplementary file 1 [file nutrients-15-02110-s001.zip › nutrients-2282952-supplementary.pdf]

Table S1. Search strategies

Medline via PUBMED
("goats"[MeSH Terms] OR "goats"[All Fields] OR "goat"[All Fields]) AND ("milk, human"[MeSH Terms] OR ("milk"[All Fields] AND "human"[All Fields]) OR "human milk"[All Fields] OR "milk"[All Fields] OR "milk"[MeSH Terms]) AND ("infant formula"[MeSH Terms] OR ("infant"[All Fields] AND "formula"[All Fields]) OR "infant formula"[All Fields]) AND (Perinat* OR neonat* OR newborn* OR infan* OR bab* OR toddler* OR boy* OR girl* OR kid* OR school*age OR juvenil* OR under*age* OR teen* OR minor* OR pubescen* OR adolescen* OR child[mh] OR child* OR pediatrics[mh] OR pediatric* OR paediatric*) AND ("perinat*"[All Fields] OR "neonat*"[All Fields] OR "newborn*"[All Fields] OR "infan*"[All Fields] OR "bab"[All Fields] OR "toddler*"[All Fields] OR "boy"[All Fields] OR "girl*"[All Fields] OR "kid"[All Fields] OR "juvenil*"[All Fields] OR "under age*"[All Fields] OR "teen*"[All Fields] OR "minor*"[All Fields] OR "pubescen*"[All Fields] OR "adolescen*"[All Fields] OR "child"[MeSH Terms] OR "child*"[All Fields] OR "pediatrics"[MeSH Terms] OR "pediatric*"[All Fields] OR "paediatric*"[All Fields]) AND ("randomized controlled trial"[Publication Type] OR "controlled clinical trial"[Publication Type] OR "randomized"[Title/Abstract] OR "placebo"[Title/Abstract] OR "clinical trials as topic"[MeSH Terms] OR "randomly"[Title/Abstract] OR "trial"[Title]) NOT ("animals"[MeSH Terms] NOT "humans"[MeSH Terms])
**CENTRAL**
([mh goats] OR goats OR goat) AND ([mh "milk, human"] OR (milk AND human) OR "human milk" OR milk OR [mh milk]) AND ([mh "infant formula"] OR (infant AND formula) OR "infant formula") AND (Perinat* OR neonat* OR newborn* OR infan* OR bab* OR toddler* OR boy* OR girl* OR kid* OR school*age OR juvenil* OR under*age* OR teen* OR minor* OR pubescen* OR adolescen* OR [mh child] OR child* OR [mh pediatrics] OR pediatric* OR paediatric*) AND (perinat* OR neonat* OR newborn* OR infan* OR bab OR toddler* OR boy OR girl* OR kid OR juvenil* OR ("under" NEXT age*) OR teen* OR minor* OR pubescen* OR adolescen* OR [mh child] OR child* OR [mh pediatrics] OR pediatric* OR paediatric*) AND ("randomized controlled trial":pt OR "controlled clinical trial":pt OR randomized:ti,ab OR placebo:ti,ab OR [mh "clinical trials as topic"] OR randomly:ti,ab OR trial:ti) NOT ([mh animals] NOT [mh humans])
**EMBASE**
(goats/exp OR goats OR goat) AND ('milk, human'/exp OR (milk AND human) OR 'human milk' OR milk OR milk/exp) AND ('infant formula'/exp OR (infant AND formula) OR 'infant formula') AND (Perinat* OR neonat* OR newborn* OR infan* OR bab* OR toddler* OR boy* OR girl* OR kid* OR school*age OR juvenil* OR under*age* OR teen* OR minor* OR pubescen* OR adolescen* OR child/exp OR child* OR pediatrics/exp OR pediatric* OR paediatric*) AND (perinat* OR neonat* OR newborn* OR infan* OR bab OR toddler* OR boy OR girl* OR kid OR juvenil* OR 'under age*' OR teen* OR minor* OR pubescen* OR adolescen* OR child/exp OR child* OR pediatrics/exp OR pediatric* OR paediatric*) AND (term:it OR term:it OR randomized:ti,ab OR placebo:ti,ab OR 'clinical trials as topic'/exp OR randomly:ti,ab OR trial:ti) NOT (animals/exp NOT humans/exp)
